# Improved Child Mental Health Following Brief Relationship Enhancement and Co-Parenting Interventions During the Transition to Parenthood

**DOI:** 10.3390/ijerph17030766

**Published:** 2020-01-25

**Authors:** Lianne M. Tomfohr-Madsen, Gerald F. Giesbrecht, Joshua W. Madsen, Anna MacKinnon, Yunying Le, Brian Doss

**Affiliations:** 1 Department of Psychology, University of Calgary, Calgary, AB T2N 1N4, Canada; ggiesbre@ucalgary.ca (G.F.G.); jmadsen@ucalgary.ca (J.W.M.); anna.mackinnon@ucalgary.ca (A.M.); 2 Alberta Children’s Hospital Research Institute (ACHRI), Calgary, AB T3B 6A8, Canada; 3 Department of Pediatrics, University of Calgary, Calgary, AB T2N 1N4, Canada; 4 Department of Community Health Sciences, University of Calgary, Calgary, AB T2N 1N4, Canada; 5 Department of Psychology, University of Miami, Miami, FL 33124, USA; yunying.le.annie@gmail.com (Y.L.); bdoss@miami.edu (B.D.)

**Keywords:** relationship therapy, co-parenting, intervention, randomized controlled trial, temperament, externalizing, internalizing, intergenerational

## Abstract

The transition to parenthood has been identified as a significant relationship stressor. Many couples report declines in relationship satisfaction and difficulty with individual stress and co-parenting—problems that have been associated with both child temperament as well as emotional and behavioral problems. Several parenting and relationship interventions have been developed to buffer against these difficulties. In the current study, we report secondary analyses of a randomized controlled trial of brief (6-h) interventions that focused on improving either relationship satisfaction or co-parenting, delivered during pregnancy and the early postpartum period. In this trial, 90 opposite-sex couples (180 participants), who were pregnant with their first child, and were assessed as being at high risk for declines in relationship satisfaction, were randomized to receive either (1) a relationship intervention, (2) a co-parenting intervention, or (3) an information control. At 12 months postpartum, couples who received either the relationship or co-parenting intervention rated their infants as having lower negative emotionality and as having fewer externalizing symptoms compared to the information-only control. Lower externalizing symptoms at 12 months were, in turn, associated with reduced externalizing symptoms at 24 months postpartum. Whereas, lower ratings of child negative emotionality at 12 months were associated with reduced internalizing symptoms at 24 months postpartum. These results indicate that brief relationship or co-parenting interventions delivered during the transition to parenthood have secondary benefits for child mental health.

## 1. Introduction

A large body of research has shown that children who grow up exposed to marital conflict and low parental relationship satisfaction are at higher risk for developing internalizing and externalizing symptoms [1]. Parents’ collaborative efforts in fulfilling child-rearing responsibilities (i.e., co-parenting) [2] are also associated with child adjustment and attachment to parents above and beyond the quality of the marital relationship [3]. Moreover, co-parenting is thought to at least partially account for the link between inter-parental relationship and child outcomes [2,4,5].

The transition to parenthood has been identified as a significant relationship stressor that is associated with declines in relationship satisfaction [6,7]. A number of individual, relationship, and infant characteristics have been shown to be associated with greater relationship declines postpartum [8]. Therefore, without adequate supports, many couples experience relationship decline, exposing their infants to less favorable contexts for optimal emotional and behavioral development and placing them at higher risk for later mental health problems.

### 1.1. Parental Interventions and Child Outcomes

Harold and Sellers (2018) make the case that, while there has been “compelling scientific evidence” for decades linking parental conflict to child outcomes, the development and dissemination of relevant treatments has lagged behind [1]. Interventions designed to improve the quality of parental relationships have varied in their long-term impact on child functioning; some studies show no long-term benefits [9,10] while others suggest long-term reductions in child behavioral problems [11,12,13]. Programs focusing on co-parenting/parenting have shown more consistent positive short- and long-term impacts on child adjustment [14,15,16,17,18,19], including some evidence of positive effects on infant temperament related to regulatory ability and internalizing and externalizing symptoms in children [17,20,21,22].

### 1.2. Developmental Origins of Child Emotional and Behavioural Problems

The foundations of child development begin in-utero and throughout the first years of life. One of the earliest indicators of later child mental health is infant temperament, which refers to individual differences in emotional reactivity and regulation [23]. Negative emotionality in particular captures an aspect of temperament that includes the expression of negative emotions, including fear, anger, irritability, sadness, and distress to limitations or discomfort [24]. Negative emotionality has strong links to the later emergence of adult neuroticism and is predictive of later mental health outcomes [25,26,27].

Although there is a clear biological basis to temperament [25], changes in infant temperament are influenced not only by biological maturation but also through environmental inputs. For example, increases in infant fear over the first year are accelerated among infants whose mothers have higher levels of depressive symptoms [28]. Maternal anxiety and maternal negative emotionality have also been shown to predict increases in infant negativity over time [29]. Conversely, responsive and sensitive parenting is associated with decreases in infants’ high reactivity [30]. Drawing together findings from the animal and human literature, even in situations where infants may be biologically primed to express higher levels of negative emotionality placing them at risk for later mental health problems—this tendency can be buffered through better family functioning, higher parental sensitivity, more positive parenting, and lower levels of neglect [1,31].

### 1.3. Relationship Intervention in the Transition to Parenthood

Given that in-utero and early childhood represent critical periods of development, as well as a period when parents are at high risk for relationship decline, it makes sense that relationship-focused interventions delivered during this time frame might yield secondary benefits for child mental health. Fortunately, programs have been developed and shown to support parent relationship quality and co-parenting behaviors in the perinatal period and to prevent declines in at-risk couples [8,32]. As one example, one study randomized couples to one of three conditions: a brief co-parenting-focused intervention, a brief relationship-focused intervention, or an information-only control group [33]. Results showed that, compared to the control group, both active interventions reduced declines in relationship satisfaction over the first two years of a child’s life in women and in high-risk men. Both groups, compared to the control, also produced improvements in women’s perceptions of parenting alliance and lower maternal stress; however, the potential secondary benefits on infant/child outcomes were not investigated.

### 1.4. The Present Study

The primary aims of this secondary data analysis are to determine the effect of the interventions described above on (1) child negative emotionality and (2) internalizing and externalizing problems. Behavioral expressions of negativity increase during an infant’s first 12 months of life as their behavioral repertoire increases [34,35,36,37] and their cognitive and neurobiological maturation allow them to anticipate and act upon the environment [36]. For this reason, our analyses focus on assessment of temperament and externalizing and internalizing symptoms beginning at the 12-month postpartum assessment and extending to the 24-month postpartum assessment.

Given findings of previous studies of relationship and co-parenting interventions on child outcomes, we hypothesized that couples who received either the couple or co-parenting intervention would rate their children lower on negative emotionality and externalizing and internalizing symptoms compared to couples in the information-only control group. As the larger trial did not show significant differentiation between the two interventions compared to the information only control group on parental outcomes, we made no hypotheses about their relative strengths compared to each other. Finally, we hypothesized that lower infant negative emotionality at 12 months would be a pathway through which group differences emerge in children’s externalizing and internalizing symptoms at 24 months.

## 2. Materials and Methods

All procedures were approved by the Texas A&M University Institutional Review Board (protocol #2005-0210). 

### 2.1. Participants and Procedures

In the original study, 90 opposite-sex couples (180 individuals) who were married or cohabitating and were between 6–8 months pregnant with their first child were recruited. The sample was restricted to couples for whom one or both partners met at least one of the seven risk factors for problems in relationship adjustment during the transition to parenthood identified through a literature review (e.g., [37,38]). The seven variables identified as risk factors included: (a) Parental divorce in family of origin; (b) father-to-mother violence in the family of origin; (c) not being currently married; (d) a previous marriage; (e) reporting that they were unsure they wanted to have a baby at this time; (f) mild-to-moderate violence in the relationship as indicated by endorsing one or more items assessing physical aggression or injury (e.g., pushing, slapping); and (g) mild-to-clinical levels of depressive symptoms, as indicated by a score of 14 or greater on the Beck Depression Inventory II upon screening.

Exclusion criteria included one member of the couple not being between 18–65 years of age, if the pregnancy was not the first child for both partners, if either partner reported severe interpersonal violence (i.e., punching or more severe) in the relationship, or if either partner was unable to speak English fluently.

#### 2.1.1. Couple Characteristics

Couples were married (86%) or cohabitating (14%). At baseline, participants were on average 27.8 years old (SD = 5.00) and had been married for an average of 2.52 years (SD = 2.47). The sample was primarily non-Hispanic White (88.3%), followed by white, Hispanic (7.8%), Native American or Alaskan Native (3.3%), Asian or Pacific Islander (2.2%), and African American (1.1%). Over half of the sample reported having either a bachelor’s degree (31%) or graduate-level degree (29%). The average individual yearly income before taxes was $25,967 (SD = 1652/month).

Stratification occurred based on whether the couples’ risk factors were distal (e.g., divorce in the family of origin) or included at least one proximal risk factor (e.g., mild-to-moderate violence in the relationship). Ninety couples were then randomly assigned to one of three conditions, which included: co-parenting intervention, relationship intervention, or an information-only control (30 couples in each group). Couples were paid a total of $500 ($250 per person) for completing all assessments; no payment was provided for attending the intervention sessions.

Couples were also randomly assigned to work with one of five intervention coaches. The coaches were Ph.D. level graduate students in clinical psychology who worked with an equal number of couples in each intervention. The coaches met with each couple separately. At pre-treatment, the couples did not differ on baseline measures of relationship or individual functioning, cumulative risk factors, or most demographic variables. The only exception was that women in the co-parenting condition had higher mean total education than women in the other two groups (F(2) = 3.458, *p* = 0.04).

#### 2.1.2. Information Control Group

Couples who were randomized to the information-only control group attended a single 90-min session before the birth of the child. The information session discussed topics associated with the transition to parenthood such as budgeting, breastfeeding, etc. The couple were able to choose topics that they were most interested in and discuss those in more depth. At the end of the session the couples were offered the option of taking home handouts and pamphlets about the aforementioned topics.

#### 2.1.3. Relationship Intervention

Couples randomized to the relationship intervention participated in four 90-min sessions (6 h in total), with two sessions conducted before birth and two session conducted approximately 3.5 months after birth. The intervention was based on Integrative Behavioral Couples Therapy (IBCT [39]). In the pre-birth sessions, couples identified a “theme” for their relationship strengths and a conversation was facilitated as to how positive relationship qualities might erode over the transition to parenthood. Current relationship difficulties and problematic communication around these were also identified and the coach helped the couple to engage in problem-solving exercises for the relationship problems. Postpartum depression, anxiety, and stress were discussed, but only as they related to the romantic relationship. The two post-birth sessions focused on how the couple’s relationship “theme” had changed. The third session focused on resolving relationship problems through acceptance-focused interventions or problem-solving exercises, as appropriate. In the fourth and final session, couples were asked to reflect on how positive aspects of their relationship had changed since becoming parents and to brainstorm ways to maintain these.

#### 2.1.4. Co-Parenting Intervention

Couples randomized to the co-parenting intervention participated in four 90-min sessions (6 h in total) with two prenatal sessions and two postnatal sessions at roughly 3.5 months postpartum. The intervention was designed to address the four components of co-parenting identified by Feinberg (2003): support/undermining, joint family management, division of labor and childrearing agreement [2]. The first prenatal session was focused on having couples discuss their expectations about the transition to parenthood, particularly pertaining to common co-parenting tasks, such as expectations about the division of labor, anticipated changes to schedules, or strategies to handle child rearing disagreements. In the second prenatal session, the coach encouraged the couple to make a “co-parenting plan” to help operationalize their expectations into a detailed behavior plan, including anticipated obstacles of implementing the plan. The couple also discussed the potential impacts of postpartum depression, anxiety, and stress on the co-parenting (but not romantic) relationship. During the first postnatal session, the couple revised the co-parenting plan and targeted problem-solving techniques were incorporated if needed. In the final session, couples worked together to create a co-parenting plan for the remainder of their child’s first year. For example, couples came up with ways to ensure consistency in limit setting as a way to both promote supportive (vs. undermining) co-parenting behavior and increase the likelihood of more effective management of interactional patterns. A more detailed description of the interventions is provided in the main outcome paper.

#### 2.1.5. Adherence

A detailed description of adherence coding can be found in the original randomized controlled trial (RCT) manuscript [33]. Briefly, adherence was rated as “excellent” across conditions, defined as covering all major and minor points of the respective interventions.

### 2.2. Measures

All participants completed assessment packages upon study entry and at 1, 3, 6, 12, 18, and 24 months postpartum. The baseline and 12-month assessments were completed in person while the other assessments were completed by mail. Individual packages were mailed to each partner to ensure their confidentiality. Temperament measures were collected at the 3-, 6-, and 12-month postpartum time points. Information about child externalizing and internalizing problems were collected at the 12-, 18-, and 24-month postpartum time points.

#### 2.2.1. Demographic and Relationship Information

At the baseline assessment, information was collected about the highest level of education, annual household income, relationship status, previous marriages, and family of origin information (i.e., parental divorce, parental violence).

#### 2.2.2. Infant Behavior Questionnaire—Revised (IBQ-R)

The IBQ-R [40] is a 191-item, multi-dimensional, parent-report measure of infant temperament. In the current study, only the 32 items of the Distress to Limitations and Fear scales were administered. These two scales are part of the Negative Emotionality composite of the IBQ-R, therefore scores from the two scales were combined into one average score. Parents reported their observations of specific infant behaviors in the previous two weeks using a 7-point Likert scale that ranged from “never” to “always.” Scores can range from 1–7 with higher scores reflecting more negative emotionality. Reliability and validity of the IBQ-R has been supported by a very large literature (e.g., Parades and Leerkes, 2008 [41]).

#### 2.2.3. Infant–Toddler Social and Emotional Assessment (ITSEA)

The ITSEA is a parent-report questionnaire assessing social-emotional problems and competencies of children ranging from 12 to 36 months of age. Parents completed a total of 166 items. The respondent rates each item on a 3-point scale (0 = not true/rarely, 1 = somewhat true/sometimes, 2 = very true/always). For certain items a respondent may also respond “N” which means no opportunity to observe the behavior. This study focused on the domains of externalizing and internalizing problems. Externalizing problems was composed of 24 items, which yielded three subscales (ranges 0–2) that included activity/impulsivity, aggression defiance, and peer aggression. The externalizing score was the mean of these three subscales, scores can range from 0–2 with higher scores reflecting more externalizing symptoms. The ITSEA domain of internalizing problems was composed of 30 items, which yielded four subscales (ranges 0–2) that included depression/withdrawal, general anxiety, separation distress, and inhibition to novelty [42,43]. The internalizing score was the mean of these four subscales, scores can range from 0–2 with higher scores reflecting more internalizing symptoms. The validity of the ITSEA has been established through associations between independent evaluator ratings and parental ratings of child behavior problems, temperament, and parental distress [42].

### 2.3. Statistical Analysis

Mother and father ratings of their child were highly correlated and there were no significant parental gender differences on ratings of infant temperament, internalizing, or externalizing symptoms at any of the time points (*p* > 0.05). Therefore, aggregate scores were created for the outcomes of negative emotionality, internalizing, and externalizing symptom by averaging parent ratings at each time point. The direct and indirect effects of the interventions on negative emotionality, externalizing, and internalizing symptoms were investigated in the steps described below. Candidate mediators (indirect effects) of 24-month externalizing and internalizing symptoms that were investigated included 12-month negative emotionality, externalizing symptoms, and internalizing symptoms.

#### 2.3.1. Mediation Analysis in Randomized Controlled Trials

In a basic mediation model, an indirect effect is represented by a path from treatment to outcome via a mediator (calculated as the product of paths a and b) and the direct effect is represented by the c’ path, after controlling for the a and b paths. An indirect or mediating effect of an independent variable, in this case treatment, can still be present even if there is no direct effect [44]. This may occur when, for example, an intervention has an effect on proximal outcomes and those more proximal outcomes have effects on distal outcomes. In the context of our RCT, an indirect effect (a*b path) represents the proportion of the intervention effect on outcomes at 24 months through the effects of treatments on outcomes at 12 months [45].

The mediation analyses were conducted in several steps. First, the effects of the intervention on each of the potential mediators (e.g., 12-month negative emotionality, internalizing, and externalizing) were examined using basic ANOVAs in Mplus 8.1(Muthén & Muthén, Los Angeles, CA, USA). The effects of the intervention on 24-month outcomes were also examined. Since a variable can only be a mediator of treatment if there is a significant effect (*p* < 0.05) of treatment on the mediator (a path), the mediation models were only fitted to variables that were significantly associated with the treatment [44]. Next, mediation models were conducted to estimate the direct and indirect effects of intervention on outcomes with regression-based path analyses in Mplus 8.1 using bias corrected 95% bootstrapped confidence intervals (CI) with 10,000 resamples. This approach accounts for the non-normality of the sampling distribution of the indirect effects [44]. Indirect effects are indicated when the CI does not contain zero [46]. As described by Hayes et al. mediation analysis with a multi-categorial independent variable were specified with indicator coding such that each intervention was compared to the information only control group [47]. Intent-to-treat analyses were conducted in line with CONSORT guidelines (i.e., all participants who were randomized were included) [48].

#### 2.3.2. Missing Data

Missing values analysis indicated 16.02% of the child data points were missing at any point, and covariance coverage for path analyses ranged from 0.80 to 1.00. A non-significant Little’s MCAR test suggests that data are likely missing completely at random (χ^2^ = 42.75, *p* = 0.61). Missing data was handled using full information maximum likelihood (FIML), which produces unbiased parameter estimates and standard errors [49].

## 3. Results

Results of the ANOVA showed that there were significant group differences on at 12-months on negative emotionality and externalizing symptoms. Compared to the information only control group both the couples therapy and co-parenting interventions had lower negative emotionality and lower externalizing symptoms. There were no other significant differences between the groups (see Table 1 and Table 2).

Univariable regression analyses of intervention effects on potential mediators and outcomes identified two potential meditators, specifically, compared to infants in the control group, infants in both intervention were rated as having lower negative emotionality (relationship intervention: *B* = −0.315, *p* = 0.038; co-parenting intervention: *B* = −0.445, *p* = 0.003) and lower externalizing symptoms (relationship intervention: *B* = −0.093, *p* = 0.021; co-parenting intervention: *B* = −0.091, *p* = 0.021) at 12-months postpartum. In terms of outcomes, there were no significant direct effects of either intervention on child internalizing or externalizing symptoms at 24 months postpartum.

### 3.1. Mediation Analyses

#### 3.1.1. 24-Month Externalizing

**Figure 1:**
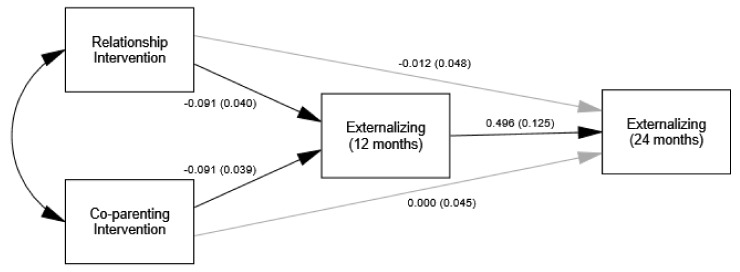
Model estimates (SE) for effects of intervention on reduced negative emotionality at 12-months and reduced infant internalizing symptoms at 24-months. Single-headed black arrows represent significant paths. Single-headed gray arrows represent non-significant paths.

The direct and indirect effects of each of the tested models are presented in Table 3. The indirect effect of intervention group through negative emotionality on 24-month externalizing symptoms was not significant for either the couple or co-parenting interventions. However, the indirect effects of group through 12-month externalizing on 24-month externalizing were significant for both couple and co-parenting interventions. As shown in Figure 1, intervention was associated with decreased 12-month externalizing which was subsequently associated with decreased 24-month externalizing.

#### 3.1.2. 24-Month Internalizing

**Figure 2:**
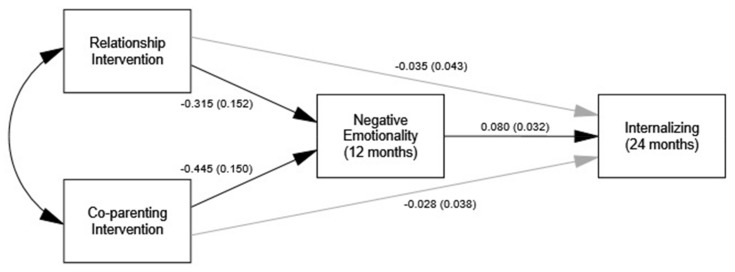
Model estimates (SE) for effects of intervention on reduced externalizing symptoms at 12-months and reduced infant externalizing symptoms at 24-months. Single-headed black arrows represent significant paths. Single-headed gray arrows represent non-significant paths.

The indirect effect of group through negative emotionality on 24-month internalizing was significant for both the couple and co-parenting interventions (see Table 4). As shown in Figure 2, intervention was associated with decreased 12-month negative emotionality that was subsequently associated with decreased 24-month internalizing.

## 4. Discussion

Many couples perceive the transition to parenthood as stressful and experience declines in relationship satisfaction during this time period [6,7]. Fortunately, programs have been developed that mitigate declines in relationship adjustment and decrease parental stress, especially in high-risk couples [33]. The results of this study showed that brief relationship interventions in the transition to parenthood conferred secondary benefits to children, such that couples who received either the co-parenting or relationship intervention in pregnancy and the early postpartum period rated their infants as having lower negative emotionality and fewer externalizing symptoms at 12 months postpartum.

Although no statistically significant direct treatment effects were observed on child functioning at 24 months, both the relationship and co-parenting interventions were indirectly associated with reduced internalizing or externalizing symptoms at 24 months through improvements in child functioning at 12 months postpartum. These findings suggest that the brief (4-session) intervention conducted during the perinatal period had a long-lasting impact on child outcomes through an influence on infant functioning at 12 months postpartum. The findings build upon an expanding area of research showing that improving parental relationship quality and co-parenting has secondary benefits for children that may reduce the likelihood of later mental health problems. Our findings are particularly encouraging as they show that these benefits are possible through brief interventions. The indirect effects findings also raise questions about the “dose” of the intervention required to have long-term child impact and whether follow-up or booster sessions may be helpful in order to enhance the impact of the intervention on later child mental health outcomes.

The findings raise the question of what intervention works best for whom? Co-parenting and relationship satisfaction have both been shown to be modifiable during the transition to parenthood, and both appear to be reciprocally linked during the early parenting years both longitudinally and on a daily basis in two parent families, so change in one can potentially have effects on the other [34,50,51,52]. However, co-parenting interventions may be more attractive than other types of therapy interventions to parents and may engender greater buy in, from fathers in particular, who describe parenting as important [33]. The potentially greater appeal to fathers is important as men are generally less amenable to help-seeking than women, and this has been observed for couple-based interventions specifically [53]. Additionally, work in other populations has shown that co-parenting is likely a link between relationship satisfaction and child adjustment [2,13,54]. Co-parenting interventions may be easier to train for delivery by para-professionals, and thus improve accessibility and cost-effectiveness. In real world settings, it may also be important to blend these intervention types depending on the specific needs of the couple. Future research is needed to determine under which circumstances a co-parenting or relationship intervention is most indicated, and whether a combined approach yields greater benefit for some couples.

### Psychological and Physiological Mechanisms

The intervention described began in pregnancy and extended into the early postpartum period. This raises questions about the potential mechanisms by which perinatal interventions may affect child development outcomes. On a physiological level, it is well-known that stress-related hormones, such as cortisol, cross the placenta and blood brain barrier, resulting in “programming” of offspring stress physiology and behavior. Furthermore, the hypothalamic-pituitary-adrenal axis is highly responsive to social relationships [55]. Positive social relationships can buffer the effects of maternal stress on child outcomes by reducing physiological stress responses in mothers during pregnancy and in infants postnatally [56,57]. In addition to physiological mechanisms, perceived support from a partner is associated with reduced depression in mothers during pregnancy and improved maternal–infant interaction postnatally, [57] suggesting that modeling and quality of care may be additional psychological mechanisms by which improvements in parental relationships contribute to improved child outcomes.

In a theoretical model linking interparental conflict to youth psychopathology, conflict is theorized to be linked to children’s neurobiological and psychophysiological arousal systems in addition to children’s cognitive and emotional processing, leading to increased risk of child psychopathology [1]. An implication of this theorized pathway is that children exposed to parental conflict will also have worse health outcomes. Conversely, brief therapy interventions delivered to expectant couples have the potential to improve both the mental and physical health profiles of the children. Although research in this area is sparse, in women who received Family Foundation (FF), a transition to parenthood program to improve co-parenting quality, FF buffered the impact of maternal mental health on birth weight and hospital stay length [58], suggesting that infants exposed to the intervention were born healthier. The long-term potential for caregiver relationship interventions to improve child health is an important future area of research.

## 5. Limitations

There are several important limitations to consider when interpreting the results of this study. First, child outcomes were assessed solely through parental reports. Parental reports have been shown to be reliable in intervention studies and to yield comparable effect sizes to clinical ratings [59]; however, parental reports may also have been influenced by the intervention received. Another possible interpretation of these findings is that instead of improving objective child outcomes, the interventions instead improved parental perceptions of their children, a limitation that could be overcome with use of objective assessments of child behavior in future studies [13]. Nevertheless, we note that improvement in parent perception of their children may be accompanied by improvement in perceived parent competence, [60] which is likely to have downstream positive effects on behavior. Additionally, the mechanisms of change driving the effects were not evaluated and so it is unclear if the intervention influenced child development through relationship factors or changes in parental mood and/or stress, each of which have been associated with parent–child interactions. Previous results from this RCT have shown that both active interventions lead to less decline in relationship satisfaction and that women experienced less perceived stress during the transition to parenthood, which may have been the mechanism of change through which child mental health was influenced. Future research with larger sample sizes should investigate these mechanisms of change. Finally, the study was also limited by a relatively small sample size, including only 30 couples per intervention group, which limited power to detect small differences in outcomes between the two active interventions.

## 6. Implications and Future Directions

Notably, the interventions tested in this trial were only four sessions in length. Despite being brief, these interventions had significant impact on child functioning that persisted for up to two years postpartum in addition to relationship and parenting benefits. The results of this study suggest that brief and low resource interventions designed to improve new parents’ relationship satisfaction have the potential to yield secondary effects on their child’s health. The brevity of the interventions makes their implementation and use very feasible for both administrators and parents in the perinatal period. These findings directly align with a recent strategic patient-oriented research (SPOR) initiative that found that new parents (conception to age 2) rated research aimed to improve relationship satisfaction and parenting confidence in the transition to parenthood as a high priority [61]. Given that child mental health problems are prevalent and rising, and that the majority of families do not have access to evidence-based treatment options, delivery of brief relationship interventions provide a potential pathway for prevention and could promote child health.

For the wide dissemination necessary to reach new parents in need of relationship intervention, e-health options are a potential avenue for further exploration [62]. In fact, results from two studies found that parents reported a preference for online formats compared to face-to-face when receiving parenting interventions [63,64]. Meta-analyses suggest that that parenting-based e-health interventions yield positive shifts in parenting and produce change in child outcomes, with moderate effect sizes [65] and recent evidence suggests a similar outcome for online couples therapy intervention [13].

A final consideration is that these findings are building on an accumulating literature showing that couples interventions are cost-effective and have associated secondary gains. Previous studies have shown that mental health and medical service utilization decrease following couple therapy, particularly in high utilizers [66,67,68]. Additionally, in the case of family therapy, secondary declines in service utilization have been observed even in individuals who did not participate directly in therapy [69]. Results from this study suggests that intervention delivered in pregnancy has a secondary benefit of decreasing negative emotionality and child externalizing symptoms, even though child behavior was not a direct focus of treatment. Interventions delivered in pregnancy have long lasting benefits and are theorized to be more cost effective than those delivered later in life [70,71,72]. Findings from this study suggest that brief interventions targeting couple satisfaction and co-parenting may be added to the list of cost-effective treatments that improve child functioning.

## 7. Conclusions

These findings suggest that delivering low intensity, relationship-enhancing interventions during the transition to parenthood has the potential to meet parents’ identified needs and confers secondary benefit to their children. Future research should continue to work to identify for whom relationship versus co-parenting interventions are most effective and to investigate modalities that can reach the largest number of couples in a cost-effective way in order to improve parental and child outcomes.

## Figures and Tables

**Table 1:** Estimated marginal means and standard errors on temperament, externalizing, and internalizing variables per group.

Treatment Group	12-Month, Mean (Standard Error)	24-Month, Mean (Standard Error)
Negative Emotionality		
Co-parenting	2.720 (0.102)	-
Couples	2.849 (0.104)	-
Information Only	3.165 (0.109)	-
Internalizing Symptoms		
Co-parenting	0.341 (0.027)	0.450 (0.029)
Couples	0.379 (0.028)	0.451 (0.030)
Information Only	0.417 (0.028)	0.509 (0.026)
Externalizing Symptoms		
Co-parenting	0.371 (0.023)	0.429 (0.030)
Couples	0.369 (0.025)	0.415 (0.032)
Information Only	0.462 (0.031)	0.478 (0.037)

**Table 2:** Pairwise Comparison of Intervention Effects on 12- and 24- month variables.

Condition Comparisons	Mean Difference (Standard Error)	*p*-Value
12-month Negative Emotionality		
Control versus co-parenting	0.445 (0.149)	0.003
Control versus couple	0.315 (0.151)	0.036
Co-parenting versus couple	−0.129 (0.146)	0.375
12-month Externalizing		
Control versus co-parenting	0.091 (0.039)	0.019
Control versus couple	0.093 (0.040)	0.020
Co-parenting versus couple	0.002 (0.034)	0.943
12-month Internalizing		
Control versus co-parenting	0.076 (0.039)	0.050
Control versus couple	0.038 (0.039)	0.329
Co-parenting versus couple	−0.038 (0.039)	0.328
24-month Externalizing		
Control versus co-parenting	0.050 (0.048)	0.297
Control versus couple	0.063 (0.049)	0.199
Co-parenting versus couple	0.013 (0.044)	0.763
24-month Internalizing		
Control versus co-parenting	0.059 (0.038)	0.125
Control versus couple	0.058 (0.040)	0.144
Co-parenting versus couple	−0.001 (0.041)	0.981

Note: Based on estimated marginal means.

**Table 3:** Mediation pathways of intervention groups predicting 24-month externalizing symptoms.

Externalizing at 12-Months as Mediator.			
	Estimate	LL 95% CI	UP 95%CI
*a* Path—intervention effects on 12-month externalizing			
Couple therapy	−0.091	−0.172	−0.014
Co-parenting	−0.091	−0.169	−0.016
*b* Path—12-month externalizing effect on 24-month externalizing			
12-month externalizing	0.496	0.270	0.751
*c’* Paths—direct effects of intervention on 24-month externalizing			
Couple therapy	−0.012	−0.108	0.080
Co-parenting	0.000	−0.092	0.086
a*b Paths—mediation pathway through 12-month externalizing			
Couple therapy	−0.045	−0.095	−0.008
Co-parenting	−0.045	−0.089	−0.011
3.2 Negative emotionality at 12-months as mediator			
*a* Paths—intervention effects on 12-month negative emotionality			
Couple therapy	−0.315	−0.613	−0.020
Co-parenting	−0.445	−0.742	−0.149
*b* Path—12-month negative emotionality effect on 24-month externalizing			
12-month negative emotionality	0.039	−0.024	0.100
*c’* Paths—direct effects of intervention on 24-month externalizing			
Couple therapy	−0.052	−0.151	0.042
Co-parenting	−0.134	−0.092	0.062
a*b Paths—mediation pathway through 12-month negative emotionality			
Couple therapy	−0.012	−0.048	0.004
Co-parenting	−0.017	−0.060	0.007

Note: 95% confidence intervals (CI) derived from bias corrected bootstrapped models with 10,000 resamples; LL—lower confidence intervals; UP—upper confidence intervals.

**Table 4:** Mediation pathways of intervention groups predicting 24-month internalizing symptoms through 12-month negative emotionality.

	Estimate	LL 95% CI	UP 95%CI
*a* Paths—intervention effects on 12-month negative emotionality			
Couple therapy	−0.315	−0.613	−0.020
Co-parenting	−0.445	−0.742	−0.149
*b* Path—12-month negative emotionality effect on 24-month internalizing			
12-month negative emotionality	0.080	0.018	0.141
*c’* Paths—direct effects of intervention on 24-month internalizing			
Couple therapy	−0.035	−0.121	0.048
Co-parenting	−0.028	−0.104	0.044
a*b Paths—mediation pathway through 12-month negative emotionality			
Couple therapy	−0.025	−0.070	−0.001
Co-parenting	−0.035	−0.085	−0.008

Note: 95% confidence intervals (CI) derived from bias corrected bootstrapped models with 10,000 resamples. LL—lower confidence intervals; UP—upper confidence intervals.

## References

[1] Harold G.T., Sellers R. (2018). Annual Research Review: Interparental conflict and youth psychopathology: An evidence review and practice focused update. J. Child Psychol. Psychiatry.

[2] Feinberg M.E. (2003). The Internal Structure and Ecological Context of Coparenting: A Framework for Research and Intervention. Parenting.

[3] Teubert D., Pinquart M. (2010). The Association Between Coparenting and Child Adjustment: A Meta-Analysis. Parenting.

[4] Parkes A., Green M., Mitchell K. (2019). Coparenting and parenting pathways from the couple relationship to children’s behavior problems. J. Fam. Psychol..

[5] Camisasca E., Miragoli S., Blasio P., Feinberg M. (2019). Co-parenting Mediates the Influence of Marital Satisfaction on Child Adjustment: The Conditional Indirect Effect by Parental Empathy. J. Child Fam. Stud..

[6] Doss B.D., Rhoades G.K., Stanley S.M., Markman H.J. (2009). The effect of the transition to parenthood on relationship quality: An 8-year prospective study. J. Pers. Soc. Psychol..

[7] Mitnick D.M., Heyman R.E., Smith Slep A.M. (2009). Changes in relationship satisfaction across the transition to parenthood: A meta-analysis. J. Fam. Psychol..

[8] Doss B.D., Rhoades G.K. (2017). The transition to parenthood: Impact on couples’ romantic relationships. Curr. Opin. Psychol..

[9] Cowan P.A., Cowan C.P., Pruett M.K., Pruett K., Wong J.J. (2009). Promoting Fathers’ Engagement With Children: Preventive Interventions for Low-Income Families. J. Marriage Fam..

[10] Gattis K.S., Simpson L.E., Christensen A. (2008). What About the Kids? Parenting and Child Adjustment in the Context of Couple Therapy. J. Fam. Psychol..

[11] Bodenmann G., Cina A., Ledermann T., Sanders M.R. (2008). The efficacy of the Triple P-Positive Parenting Program in improving parenting and child behavior: A comparison with two other treatment conditions. Behav. Res. Ther..

[12] Zemp M., Milek A., Cummings E., Cina A., Bodenmann G. (2016). How Couple- and Parenting-Focused Programs Affect Child Behavioral Problems: A Randomized Controlled Trial. J. Child Fam. Stud..

[13] Doss B.D., Roddy M.K., Llabre M.M., Georgia Salivar E., Jensen-Doss A. (2019). Improvements in Coparenting Conflict and Child Adjustment Following an Online Program for Relationship Distress. J. Fam. Psychol..

[14] Barton A.W., Beach S.R.H., Kogan S.M., Stanley S.M., Fincham F.D., Hurt T.R., Brody G.H. (2015). Prevention Effects on Trajectories of African American Adolescents’ Exposure to Interparental Conflict and Depressive Symptoms. J. Fam. Psychol..

[15] Cummings E.M., Faircloth W.B., Mitchell P.M., Cummings J.S., Schermerhorn A.C. (2008). Evaluating a Brief Prevention Program for Improving Marital Conflict in Community Families. J. Fam. Psychol..

[16] Cowan C.P., Cowan P.A., Barry J. (2011). Couples’ Groups for Parents of Preschoolers: Ten-Year Outcomes of a Randomized Trial. J. Fam. Psychol..

[17] Feinberg M.E., Kan M.L. (2008). Establishing Family Foundations: Intervention Effects on Coparenting, Parent/Infant Well-Being, and Parent-Child Relations. J. Fam. Psychol..

[18] Faircloth W.B., Schermerhorn A.C., Mitchell P.M., Cummings J.S., Cummings E.M. (2011). Testing the long-term efficacy of a prevention program for improving marital conflict in community families. J. Appl. Dev. Psychol..

[19] Jones D.E., Feinberg M.E., Hostetler M.L., Roettger M.E., Paul I.M., Ehrenthal D.B. (2018). Family and Child Outcomes 2 Years After a Transition to Parenthood Intervention. Fam. Relat..

[20] Feinberg M., Jones D., Hostetler M., Roettger M., Paul I., Ehrenthal D. (2016). Couple-Focused Prevention at the Transition to Parenthood, a Randomized Trial: Effects on Coparenting, Parenting, Family Violence, and Parent and Child Adjustment. Prev. Sci..

[21] Feinberg M.E., Jones D.E., McDaniel B.T., Liu S., Almeida D. (2019). Chapter II: New Fathers’ and Mothers’ Daily Stressors and Resources Influence Parent Adjustment and Family Relationships. Monogr. Soc. Res. Child Dev..

[22] Feinberg M.E., Kan M.L., Goslin M.C. (2009). Enhancing Coparenting, Parenting, and Child Self-Regulation: Effects of Family Foundations 1 Year after Birth. Prev. Sci..

[23] Rothbart M. (2007). Temperament, Development, and Personality. Curr. Dir. Psychol. Sci..

[24] Thomas A., Chess S., Birch H.G. (1968). Temperament and Behaviour Disorders in Children.

[25] Rothbart M.K., Bates J.E., Damon W., Lerner R., Eisenberg N. (2006). Temperament. Handbook of Child Psychology, Vol. 3. Social, Emotional, and Personality Development.

[26] Rothbart M.K., Derryberry D., Lamb M.E., Brown A. (1981). Development of individual differences in temperament. Advances in Developmental Psychology.

[27] Lemery-Chalfant K., Clifford S., Swann G., Kim Y. (2014). Specificity in shared genetic effects. Temperament and Child Psychopathology.

[28] Gartstein M.A., Bridgett D.J., Rothbart M.K., Robertson C., Iddins E., Ramsay K., Schlect S. (2010). A latent growth examination of fear development in infancy: Contributions of maternal depression and the risk for toddler anxiety. Dev. Psychol..

[29] Malatesta C.Z., Haviland J.M., Izard C.E., Read P.B. (1982). Measuring change in infant emotional expressivity: Two approaches applied in longitudinal investigation. Measuring Emotions in Infants and Children: Based on Seminars Sponsored by the Committee on Social and Affective Development During Childhood of the Social Science Research Council.

[30] Belsky J., Fish M., Isabella R. (1991). Continuity and Discontinuity in Infant Negative and Positive Emotionality—Family Antecedents and Attachment Consequences. Dev. Psychol..

[31] Barlow D.H., Ellard K.K., Sauer-Zavala S., Bullis J.R., Carl J.R. (2014). The Origins of Neuroticism. Perspect. Psychol. Sci..

[32] Pilkington P., Rominov H., Brown H.K., Dennis C.-L. (2019). Systematic review of the impact of coparenting interventions on paternal coparenting behavior. J. Adv. Nurs..

[33] Doss B.D., Cicila L.N., Hsueh A.C., Morrison K.R., Carhart K. (2014). A randomized controlled trial of brief coparenting and relationship interventions during the transition to parenthood. J. Fam. Psychol..

[34] Denham S.A., Lehman E.B., Moser M.H., Reeves S.L. (1995). Continuity and change in emotional components of infant temperament. Child Study J..

[35] Brooker R.J., Buss K.A., Lemery-Chalfant K., Aksan N., Davidson R.J., Goldsmith H.H. (2013). The development of stranger fear in infancy and toddlerhood: Normative development, individual differences, antecedents, and outcomes. Dev. Sci..

[36] Sroufe L.A. (1995). Emotional Development: The Organization of Emotional Life in the Early Years.

[37] Halford W.K., Price J., Kelly A.B., Bouma R., Young R.M. (2001). Helping the female partners of men abusing alcohol: A comparison of three treatments. Addiction.

[38] Petch J.F., Halford W.K., Creedy D.K., Gamble J. (2012). A randomized controlled trial of a couple relationship and coparenting program (Couple CARE for Parents) for high- and low-risk new parents. J. Consult. Clin. Psychol..

[39] Jacobson N.S. (1996). Integrative Couple Therapy: Promoting Acceptance and Change.

[40] Gartstein M.A., Rothbart M.K. (2003). Studying infant temperament via the Revised Infant Behavior Questionnaire. Infant Behav. Dev..

[41] Parade S.H., Leerkes E.M. (2008). The reliability and validity of the Infant Behavior Questionnaire-Revised. Infant Behav. Dev..

[42] Carter A., Briggs-Gowan M., Jones S., Little T. (2003). The Infant–Toddler Social and Emotional Assessment (ITSEA): Factor Structure, Reliability, and Validity. Off. Publ. Int. Soc. Res. Child Adolesc. Psychopathol..

[43] Community-University Partnership for the Study of Children, Youth, and Families (2012). Review of the Infant-Toddler Social and Emotional Assessment (ITSEA). https://cloudfront.ualberta.ca/-/media/ualberta/faculties-and-programs/centres-institutes/community-university-partnership/resources/tools---assessment/itseajun2012.pdf.

[44] Hayes A. (2013). Introduction to Mediation, Moderation, and Conditional Process Analysis: A Regression-Based Approach.

[45] Whittle R., Mansell G., Jellema P., Windt D. (2017). Applying causal mediation methods to clinical trial data: What can we learn about why our interventions (don’t) work?. Eur. J. Pain.

[46] Hayes A.F. (2009). Beyond Baron and Kenny: Statistical mediation analysis in the new millennium. Commun. Monogr..

[47] Hayes A.F., Preacher K.J. (2014). Statistical mediation analysis with a multicategorical independent variable. Br. J. Math. Stat. Psychol..

[48] Schulz K.F., Altman D.G., Moher D. (2010). CONSORT 2010 statement: Updated guidelines for reporting parallel group randomized trials. Ann. Intern. Med..

[49] Enders C.K. (2010). Applied Missing Data Analysis. Methodology in the Social Sciences Series.

[50] Durtschi J.A., Soloski K.L., Kimmes J. (2017). The Dyadic Effects of Supportive Coparenting and Parental Stress on Relationship Quality Across the Transition to Parenthood. J. Marital Fam. Ther..

[51] Le Y., Fredman S.J., McDaniel B.T., Laurenceau J.-P., Feinberg M.E. (2019). Cross-Day Influences Between Couple Closeness and Coparenting Support Among New Parents. J. Fam. Psychol..

[52] Le Y., McDaniel B.T., Leavitt C.E., Feinberg M.E. (2016). Longitudinal Associations Between Relationship Quality and Coparenting Across the Transition to Parenthood: A Dyadic Perspective. J. Fam. Psychol..

[53] Doss B.D., Atkins D.C., Christensen A. (2003). Who’s dragging their feet? Husbands and wives seeking marital therapy. J. Marital Fam. Ther..

[54] Margolin G., Gordis E.B., John R.S. (2001). Coparenting: A Link Between Marital Conflict and Parenting in Two-Parent Families. J. Fam. Psychol..

[55] Gunnar M.R., Donzella B. (2002). Social regulation of the cortisol levels in early human development. Psychoneuroendocrinology.

[56] Thomas J.C., Letourneau N., Campbell T.S., Giesbrecht G.F., Team A.S. (2018). Social buffering of the maternal and infant HPA axes: Mediation and moderation in the intergenerational transmission of adverse childhood experiences. Dev. Psychopathol..

[57] Thomas J.C., Letourneau N., Bryce C.I., Campbell T.S., Giesbrecht G.F., Team A.P.S. (2017). Biological embedding of perinatal social relationships in infant stress reactivity. Dev. Psychobiol..

[58] Feinberg M., Jones D., Roettger M., Hostetler M.L., Sakuma K.L., Paul I.M., Ehrenthal D.B. (2016). Preventive Effects on Birth Outcomes: Buffering Impact of Maternal Stress, Depression, and Anxiety. Matern. Child Health J..

[59] Weisz J.R., Kuppens S., Ng M.Y., Eckshtain D., Ugueto A.M., Vaughn-Coaxum R., Jensen-Doss A., Hawley K.M., Krumholz Marchette L.S., Chu B.C. (2017). What Five Decades of Research Tells Us About the Effects of Youth Psychological Therapy: A Multilevel Meta-Analysis and Implications for Science and Practice. Am. Psychol..

[60] Colalillo S., Johnston C. (2016). Parenting cognition and affective outcomes following parent management training: A systematic review. Clin. Child Fam. Psychol. Rev..

[61] Bright K.S., Ginn C., Keys E.M., Brockway M.L., Tomfohr-Madsen L., Doane S., Benzies K. (2018). Study Protocol: Determining Research Priorities of Young Albertan Families (The Family Research Agenda Initiative Setting Project-FRAISE)-Participatory Action Research. Front. Public Health.

[62] Doss B.D., Benson L.A., Georgia E.J., Christensen A. (2013). Translation of Integrative Behavioral Couple Therapy to a Web-based Intervention. Fam. Process.

[63] Metzler C.W., Sanders M.R., Rusby J.C., Crowley R.N. (2012). Using Consumer Preference Information to Increase the Reach and Impact of Media-Based Parenting Interventions in a Public Health Approach to Parenting Support. Behav. Ther..

[64] Tully L.A., Piotrowska P.J., Collins D.A.J., Mairet K.S., Black N., Kimonis E.R., Hawes D.J., Moul C., Lenroot R.K., Frick P.J. (2017). Optimising child outcomes from parenting interventions: Fathers’ experiences, preferences and barriers to participation. BMC Public Health.

[65] Nieuwboer C.C., Fukkink R.G., Hermanns J.M.A. (2013). Online programs as tools to improve parenting: A meta-analytic review. Child. Youth Serv. Rev..

[66] Madsen J.W., Tomfohr-Madsen L.M., Doss B.D. (2017). The Impact of Couple Therapy on Service Utilization among Military Veterans: The Moderating Roles of Pretreatment Service Utilization and Premature Termination. Fam. Process.

[67] Georgia Salivar E.J., Rothman K., Roddy M.K., Doss B.D. (2018). Relative Cost Effectiveness of In-Person and Internet Interventions for Relationship Distress. Fam. Process.

[68] Law D.D., Crane D.R. (2000). The influence of marital and family therapy on health care utilization in a health-maintenance organization. J. Marital Fam. Ther..

[69] Crane D., Christenson J. (2012). A Summary Report of the Cost-Effectiveness of the Profession and Practice of Marriage and Family Therapy. Int. J..

[70] University CotDCaH (2010). The Foundations of Lifelong Health Are Built in Early Childhood. https://46y5eh11fhgw3ve3ytpwxt9r-wpengine.netdna-ssl.com/wp-content/uploads/2010/05/Foundations-of-Lifelong-Health.pdf.

[71] Heckman J. The Heckman Curve—The Heckman Equation. https://heckmanequation.org/resource/the-heckman-curve/.

[72] Heckman J., Pinto R., Savelyev P. (2013). Understanding the Mechanisms Through Which an Influential Early Childhood Program Boosted Adult Outcomes. Am. Econ. Rev..

